# Incidence, risk factors, and mortality impact of chronic kidney disease in middle-aged Finns: a 22-year population-based cohort study

**DOI:** 10.1186/s12882-026-04970-6

**Published:** 2026-04-14

**Authors:** Johanna Sinkko, Kati Kaartinen, Sirkka Keinänen-Kiukaanniemi, Jaakko Tuomilehto, Hannu Uusitalo, Jouko Saramies, Patrik Finne

**Affiliations:** 1 Department of Nephrology, Helsinki University Hospital and University of Helsinki, Helsinki, Finland; 2 Wellbeing Services County of South Karelia, Lappeenranta, Finland; 3 Wellbeing Services County of North Ostrobothnia Pohde, Oulu, Finland; 4 Medical Research Center, Oulu University Hospital, Oulu, Finland; 5 Department of Public Health, University of Helsinki, Helsinki, Finland; 6 SILK, Department of Ophthalmology, Faculty of Medicine and Health Technology and Tays Eye Centre, Tampere University, Tampere University Hospital, Tampere, Finland; 7 South Karelia Central Hospital, Valto Käkelän katu 1, Lappeenranta, 53130 Finland

**Keywords:** 40% eGFR decline, Chronic kidney disease, Mortality, Proteinuria

## Abstract

**Background:**

Although many studies have estimated the prevalence of chronic kidney disease (CKD) in the general population, few have assessed its cumulative incidence over a long time using both estimated glomerular filtration rate (eGFR) and albuminuria or assessed how CKD occurrence influences mortality.

**Methods:**

This prospective, population-based cohort study included 1144 Finnish participants aged 40–65 years at baseline, who were followed from 1996 to 2019. The study assessed the prevalence of CKD stages 1–5 at baseline, 10 years, and 22 years, and examined risk factors for incident CKD, defined as a ≥ 40% decline in eGFR, new-onset proteinuria, or a new kidney disease diagnosis. It also evaluated the association between incident CKD, eGFR decline, and proteinuria and mortality. Multivariable Cox proportional hazards models were used to assess risk factors of incident CKD, and the effect of eGFR decline or proteinuria on mortality.

**Results:**

At baseline, the prevalence of CKD was 3.1%. During the follow-up, incident CKD was detected in 14.1% of participants. In multivariable analysis, older age, higher body mass index, diabetes and hypertension were associated with increased risk of CKD. After adjustment for age, incident CKD was associated with 2.31-fold risk of all-cause mortality, and when evaluated separately, proteinuria and a ≥ 40% decline in eGFR were associated with 2.75-fold and 2.37-fold risks, respectively.

**Conclusion:**

Incidence of CKD is high in middle-aged Finns and is strongly associated with increased mortality.

**Supplementary Information:**

The online version contains supplementary material available at https://doi.org/10.1186/s12882-026-04970-6.

## Introduction

The prevalence of chronic kidney disease (CKD) is increasing rapidly, and CKD contributes substantially to healthcare burden and costs. According to current estimates, CKD will become the fifth leading cause of death by 2040 [1]. A recent meta-analysis [2] indicates that the global prevalence of CKD stages 1–5 is 13.0%. The prevalence is highest in low- and middle-income countries, while in Europe it is approximately 10% for stages 1–5 and 7.2% for stages 3–5. In Finland, the FINRISK study reported a 2.6% prevalence of stage 3–5 CKD in individuals aged 25–74 years in 2007 [3], while a more recent study, with a median participant age of 76 years, found a 6.3% prevalence of stage 3–4 CKD [4]. However, many epidemiological studies of CKD have relied solely on estimated GFR (eGFR), with limited information on proteinuria. In clinical practice, screening for CKD remains insufficient – even among high-risk groups [4, 5] – leading to underdiagnosis, particularly of early-stage CKD (stages 1–2). Moreover, most existing research has focused on point prevalence of CKD, while long-term incidence data on CKD in the general population are scarce.

The early recognition of CKD is crucial for slowing disease progression and preventing secondary complications, particularly cardiovascular events. Many key risk factors for CKD – such as hypertension, diabetes, and smoking – are modifiable through timely intervention. However, low awareness of CKD remains a major barrier to effective treatment adherence and follow-up [6]. Numerous studies have demonstrated that CKD is associated with increased mortality, mainly due to cardiovascular events. Multiple mechanisms contribute to the increased mortality, including systemic effects of uremic toxicity and the burden of associated comorbidities [7]. While both proteinuria and eGFR decline have been independently linked to higher mortality risk, it remains unclear which marker is more strongly associated with it.

The aims of this study were to evaluate the long-term cumulative risk and risk factors for incident CKD in the general population, and to assess the impact of proteinuria and eGFR decline on mortality risk.

## Methods

### Study design

The study population comprises participants in the Savitaipale Study, a prospective population-based cohort study conducted in South-Eastern Finland in a rural municipality with 4200 inhabitants. All 1508 residents born between 1933 and 1956 and living in Savitaipale were invited to participate in the baseline survey conducted between 1996 and 1999. Follow-up surveys were conducted at 10 years and 22 years after baseline. Data collection included structured interviews, self-administered questionnaires, clinical examinations, laboratory tests, and information retrieved from several national health registries and local medical records. The complete study design has been described in detail elsewhere [8].

All participants with available serum creatinine or urine protein measurements through study visits or measurements identified from local healthcare records were included. Additionally, participants having a diagnosis in national health registries indicating primary kidney disease were included. All data collected were pseudonymized, and the analyses were performed in a secure data environment.

### Study population

**Fig. 1:**
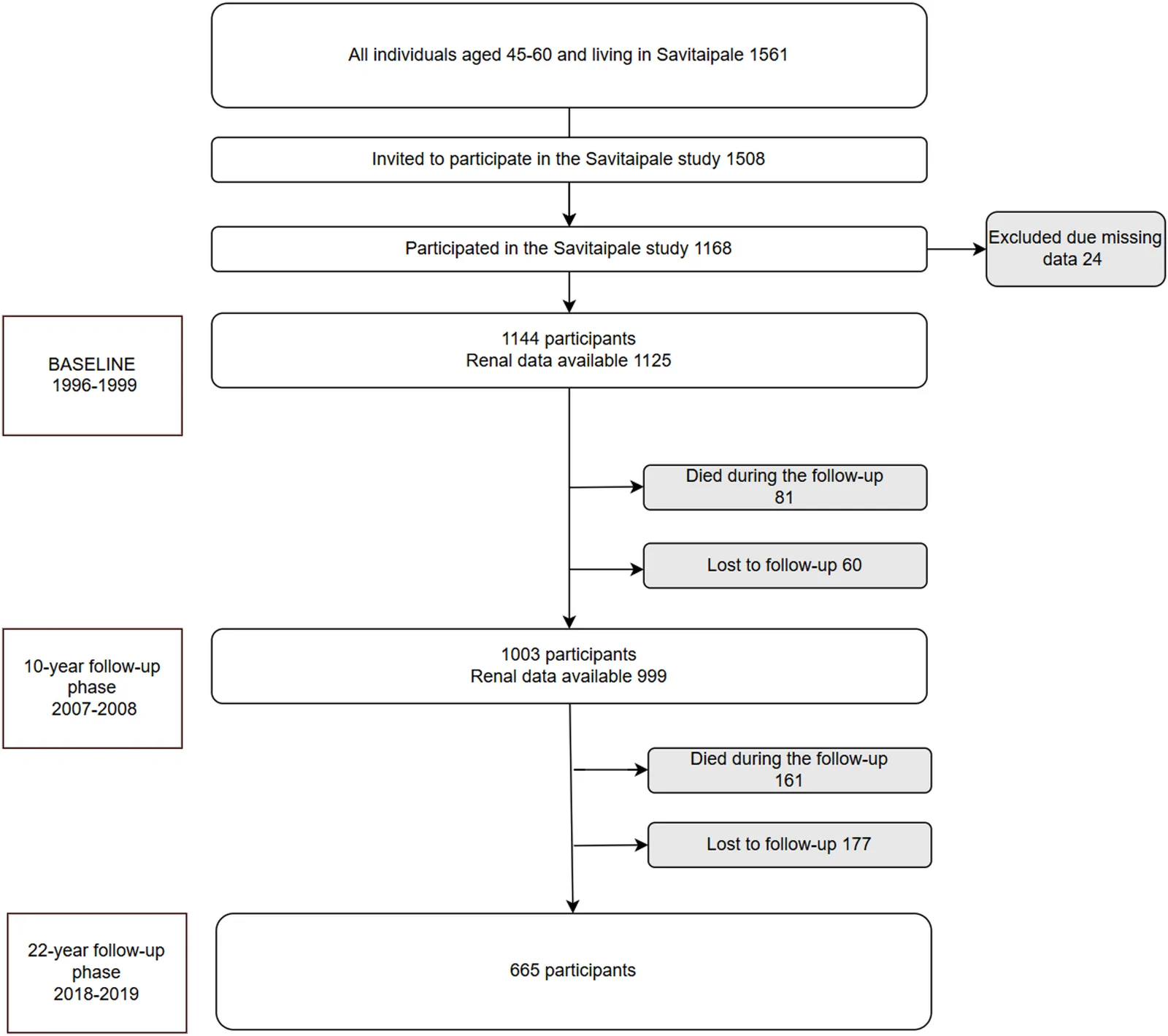
Flowchart of the study

Participants with missing data on renal function were excluded from the final study population, yielding 1144 participants. Data on eGFR, proteinuria, or a CKD diagnosis were available for 1125 participants at baseline, 999 at 10-year follow-up, and 665 at the 22-year follow-up. A flowchart illustrating the study population and follow-up is presented in Fig. 1.

### Data extraction

Clinical measurements at each time point included height, weight, waist and hip circumference, and blood pressure. Body mass index (BMI) was calculated as body weight in kilograms (kg) divided by the square of height in meters (kg/m^2^). The questionnaire assessing participants’ health status and lifestyle is included in Supplementary file 1. Laboratory tests in the original study protocol included standardized 2-hour glucose tolerance test (OGTT), fasting plasma total cholesterol (TC), high-density lipoprotein (HDL) cholesterol, triglycerides (Tg), and insulin. Low-density lipoprotein cholesterol (LDL) was calculated using the Friedewald formula: LDL(mmol/l) = TC(mmol/l) – HDL (mmol/l) – Tg(mmol/l)/2.2) [9]. The 22-year follow-up phase also included glycated hemoglobin (HbA1c) and urinary albumin-to-creatinine ratio (ACR). For the present study, serum creatinine (SCr), serum albumin and cystatin C (CysC) were analysed from stored frozen serum samples. All laboratory analyses were performed in a certified laboratory, with SCr measured using an enzymatic method. A detailed description of laboratory procedures is provided in Supplementary file 2.

Estimated GFR was calculated using the CKD-EPI-equation based on SCr [10] at all study phases and using both SCr and CysC [11] at the 22-year follow-up. According to the Kidney Disease: Improving Global Outcomes (KDIGO) clinical practice guidelines, eGFR was classified into stages 1–5 [12].

CKD-EPI creatinine equation was calculated as: 141×min(SCr/κ, 1)^α^×max(SCr/κ, 1)^−1.209^ × 0.993^Age^ ×1.018 [if female] ×1.159 [if black], where SCr is serum creatinine, κ is 0.7 for females and 0.9 for males, α is − 0.329 for females and − 0.411 for males, min indicates the minimum of SCr/κ or 1, and max indicates the maximum of SCr/κ or 1, and age is presented in years.

CKD-EPI Creatinine-Cystatin C was calculated as: 135xmin(Scr/κ, 1) ^α^xmax (SCr/κ, 1)^−0.544^ xmin(CysC/0.8, 1)^−0.323^ xmax (CysC/0.8, 1)^−0.778α^x0.9961^Age^ ×0.963[if female], where SCr is serum creatinine in mg/dl, κ is 0.7 for females or 0.9 for males, α is − 0.219 for females or − 0.144 for males, min is the minimum of SCr/κ or 1, and max is the maximum of SCr/κ or 1, CysC is standardized serum Cystatin C in mg/l, and age is presented in years.

Additional laboratory data were collected from local healthcare registers. All SCr values and urine protein determinations performed in primary care between 1996 and 2019 were included, yielding 4151 eGFR determinations for 811 participants and 4730 urine samples for 882 participants in addition to those collected during study visits. These comprised urine albumin-to-creatinine ratio (ACR), 24-hour urine protein collections (PER), timed urine collections for albumin (cU-Alb, nU-Alb), and urine dipstick tests. Urine leukocytes, erythrocytes, and bacterial cultures were also obtained. Urine dipstick results were evaluated separately to exclude samples obtained during urinary tract infection.

The study entry date for each participant was defined as the date of their first available measurements. Participants with previously confirmed CKD diagnosis, persistent proteinuria or eGFR < 60 ml/min/1.73m^2^ were classified as having CKD at study entry. For each study phase, the most recent SCr or urine test result was used. Measurements obtained between the study phases were included to define the onset of CKD if they met the CKD criteria for the first time. For participants who withdrew the study before the 22-year follow-up phase, the date of the last available laboratory value was recorded as the end of the follow-up. For deceased individuals, the date of death was recorded as the end of follow-up.

Diabetes at baseline was defined as previously confirmed diabetes or blood glucose levels diagnostic of diabetes in an OGTT (fasting plasma glucose ≥ 7.0 mmol/l or 2 h post-load glucose ≥ 11.1 mmol/l). Hypertension at baseline was defined as systolic blood pressure > 140 and/or diastolic pressure > 90 mmHg or antihypertensive medication. Smoking at baseline was defined as self-reported current or former smoking.

### Laboratory characteristics

Results of eGFR were available for 1143 participants, for whom eGFR values of 331 participants came only from the study visits, 39 had values only from healthcare records, and 773 had values from both sources. For 74 participants, only a single measurement of eGFR was available; however, none of them showed signs of kidney disease (i.e., decreased eGFR or proteinuria). For 572 participants, eGFR measurements were available at all three study phases. Urine sample results were available for 918 participants. Among them, 33 had results only from the study visits, 301 only from healthcare records, and 584 had data from both sources.

### CKD definition

CKD was defined as an eGFR < 60 ml/min/1.73m^2^, an ACR ≥3 mg/mmol (or an equivalent value by other validated methods), or the presence of an ICD-10 (The International Classification of Diseases, 10th Revision) diagnostic code indicative of CKD (E10.2, E11.2, E12.2, I12-, I13-, N00-N29, Q60-Q64, Z49-, Z99.2 and Z94.0). Incident CKD was defined by one or more of the following criteria: (I) a ≥ 40% decline in eGFR compared to the first available measurement; (II) new-onset proteinuria equivalent to ACR ≥3 mg/mmol, or (III) a new ICD-10 diagnosis indicative of CKD. A transient decline in eGFR for less than 90 days was not considered CKD. Participants who had eGFR < 60 ml/min/1.73m^2^ in study samples but ≥ 60 ml/min in subsequent healthcare registry measurements were considered not to have CKD. If the chronicity was confirmed by a second qualifying eGFR measurement ≥ 90 days after the first, the date of the initial measurement was defined as the CKD onset. For participants who had initiated dialysis, the date of dialysis initiation was considered the time of ≥ 40% eGFR decline.

Urine-based criteria for identifying CKD included any of the following findings: ACR ≥3 mg/mmol, PER ≥ 150 mg/24hours, timed cU-Alb or nU-Alb ≥ 20 µg/min, or a urinary protein dipstick test result 2 + or 3 + in the absence of a concurrent bacterial urinary tract infection. A urinary protein dipstick test result of 1 + without evidence of simultaneous bacterial infection was considered as kidney disease if the finding was confirmed by a repeated abnormal result at least 90 days later.

### Clinical endpoints

The primary endpoint was incident CKD as defined by the criteria outlined above. The secondary endpoint was all-cause mortality obtained from the National Death Register using the computerized record linkage with the Finnish personal identification number; thus, the ascertainment of vital status was complete.

### Register data

Laboratory results were collected from the medical records of Savitaipale primary care and South Karelia social district. Diagnostic codes were obtained via the Finnish Institute for Health and Welfare´s Register of Primary health care visits and the Care Register for health care. Medical reimbursement data was obtained from the Social Insurance Institution of Finland. Date and cause of death were obtained from Statistics Finland.

### Statistical analyses

All statistical analyses were performed using IBM SPSS Statistics version 29.0 (IBM Corp) and R studio 2024.1.1. Comparison between groups was performed employing the Pearson´s χ^2^ test for categorical variables and Mann-Whitney U test for continuous variables. Cox regression analysis was used to assess the predictors of incident CKD and mortality. In these models, the event was either incident CKD or death, and participants were censored at the time of loss to follow-up, or at the end of the study period, whichever occurred first. Fine and Gray competing risks regression was performed, treating death as a competing risk for incident CKD, and the crr (Competing Risks Regression) and crrs (Competing Risks Regression for Stratified Data) functions were used. For the mortality analysis, the time-dependent Cox regression procedure in SPSS was used, with a single-record structure for follow up-time. This approach is mathematically equivalent to the start-stop implementation, where the follow-up time is split at the time of the event. Incident CKD, proteinuria and eGFR decline ≥ 40% were treated in these analyses as time-dependent variables, and the exposure variables were included in separate models. The proportional hazards assumption was assessed using log-minus-log survival plots and by examining associations between partial residuals and time. Results are reported as hazard ratios (HRs) with 95% confidence intervals (CI). A two-sided *P*-value < 0.05 was considered statistically significant.

Variables that were statistically significant in age- and sex-adjusted analysis were included in the multivariable Cox regression model. However, systolic and diastolic blood pressure, fasting plasma glucose and 2-hour plasma glucose were excluded to avoid collinearity with baseline diabetes and hypertension status. Triglycerides and HDL cholesterol had statistically significant correlation with BMI, and they were excluded, as they were considered metabolic consequences of obesity and as part of the same causal pathway with BMI, rather than being independent factors.

## Results

Baseline characteristics of the cohort are presented in Table 1 and in Supplementary file 3. A total of 1144 individuals were included in the study, comprising 571 men and 573 women. The median age of participants at baseline was 51.6 years, and the median eGFR was 100 ml/min/1.73m^2^, indicating generally preserved kidney function at study entry.

**Table 1:** Baseline characteristics of participants

Characteristics	Total	CKD			*P*-value
		prior study entry	Incident CKD	No CKD	
	(*n* = 1144)	(*n* = 14)	(*n* = 161)	(*n* = 969)	
Age (years)	51.6 (46.4–58.9)	52.8 (48.9–61.5)	55.9 (48.6–61.3)	50.8 (46.0–58.7)	< 0.001
Male sex	571 (49.9%)	10 (71.4%)	77 (47.8%)	484 (49.9%)	0.618
eGFR (ml/min/1.73m^2^)	100 (92–106)	64 (52–92)	97 (89–105)	100 (93–106)	< 0.001
Missing data, n (%)	61 (5.3%)	0	6 (3.7%)	55 (5.7%)	
Smoking					0.001
Never	600 (52.8%)	7 (53.8%)	103 (64.4%)	490 (50.8%)	
Current or former	537 (47.2%)	6 (46.2%)	57 (35.6%)	474 (49.2%)	
Missing data, n (%)	7 (0.6%)	1 (7.1%)	1 (0.6%)	5 (0.5%)	
SBP (mmHg)	132 (120–147)	134 (125–141)	142 (127–157)	131 (119–145)	< 0.001
DBP (mmHg)	84 (76–92)	88 (80–95)	87 (80–94)	83 (76–91)	< 0.001
Antihypertensive medication	199 (17.4%)	6 (42.9%)	47 (29.2%)	146 (15.1%)	< 0.001
Hypertension	554 (48.4%)	10 (71.4%)	105 (65.2%)	439 (45.3%)	< 0.001
BMI (kg/m^2^)	25.8 (23.4–28.6)	26.6 (23.6–33.1)	27.0 (24.2–30.8)	25.6 (23.3–28.3)	< 0.001
Missing data, n (%)	3 (0.2%)	0	2 (0.2%)	1 (0.6%)	
OGTT					
fasting plasma glucose (mmol/l)	4.7 (4.4–5.1)	4.8 (4.4–5.1)	4.8 (4.5–5.1)	4.7 (4.4–5.1)	0.218
2 h post-load plasma glucose (mmol/l)	5.2 (4.4–6.3)	4.7 (4.2–5.9)	5.5 (4.5–7.0)	5.2 (4.3–6.2)	0.009
Missing data, n (%)	46 (4.0%)	4 (28.6%)	17 (10.6%)	25 (2.6%)	
Diabetes	78 (6.8%)	5 (35.7%)	23 (14.3%)	50 (5.2%)	< 0.001
Plasma total cholesterol (mmol/l)	5.5 (4.9–6.2)	5.4 (4.7–8.0)	5.5 (5.0–6.2)	5.6 (4.9–6.2)	0.801
Plasma HDL-cholesterol (mmol/l)	1.4 (1.2–1.7)	1.2 (1.1–1.5)	1.4 (1.1–1.7)	1.4 (1.2–1.7)	0.022
Plasma triglycerides (mmol/l)	1.1 (0.8–1.6)	1.3 (0.9–4.0)	1.3 (0.8–2.0)	1.1 (0.8–1.6)	0.011
Plasma LDL-cholesterol (mmol/l)	3.5 (2.9–4.1)	3.2 (3.0–4.0)	3.5 (2.9–4.1)	3.5 (2.9–4.1)	0.675
Missing data, n (%)	10 (0.9%)	0	3 (1.9%)	7 (0.7%)	
Serum albumin (g/l)	38 (35–40)	38 (34–40)	37 (34–39)	38 (35–40)	0.022
Missing data, n (%)	106 (9.3%)	5 (35.7%)	18 (11.2%)	83 (8.6%)	

The values are expressed as medians with interquartile ranges or numbers with percentages. *P*-values were tested between

Incident CKD and No CKD groups using the Chi-square test and the Mann-Whitney U test as appropriate. Sample sizes vary by laboratory test due to missing data

Abbreviations: CKD, chronic kidney disease; eGFR, estimated glomerular filtration rate; SBP, systolic blood pressure; DBP, diastolic

blood pressure; BMI, body mass index; OGTT, oral glucose tolerance test; HDL, high-density lipoprotein; LDL, low-density lipoprotein

### Incident CKD

Over the follow-up period, incident CKD was detected in 161 participants (14.1%), including 77 men and 84 women. Among them, 86 had isolated proteinuria, 52 experienced a ≥ 40% decline in eGFR, and 17 had both findings. Additionally, 6 participants were classified as having incident CKD solely on the basis of ICD-10 diagnostic codes.

Proteinuria was detected in 103 participants. Of these, 34 participants had proteinuria confirmed in at least two consecutive urine samples collected > 90 days apart. In 69 participants, only a single urine sample indicated proteinuria; however, 11 of these had concurrent reduced eGFR, supporting a CKD diagnosis.

Participants who developed incident CKD were older at baseline. The proportion of men was similar, but BMI, triglycerides, and the proportion of hypertension were higher in participants who later developed CKD. They also had diabetes more frequently, lower eGFR, lower HDL cholesterol and lower serum albumin at baseline. Smoking was more common among participants without incident CKD (Table 1).

Table 2 presents both unadjusted and age- and sex-adjusted hazard ratios for the association between baseline variables and incident CKD. The analysis showed that several metabolic and cardiovascular risk factors were significantly associated with increased CKD risk: higher fasting glucose, 2-hour glucose, BMI, systolic blood pressure, diastolic blood pressure, triglycerides, baseline diabetes and hypertension status were all associated with an increased risk of developing incident CKD. Conversely, lower eGFR and lower HDL cholesterol at baseline were associated with a higher risk of incident CKD.

**Table 2:** Association between risk factors and incident chronic kidney disease

Factor	Unadjusted analysis	*P*-value	Age- and sex-adjusted analysis	*P*-value	Number of events/participants
	HR (95% CI)		HR (95% CI)		
Age (years)	1.10 (1.07–1.12)*	< 0.001	N/A	N/A	161/1130
Sex (male)	1.06 (0.78–1.44)	0.731	N/A	N/A	161/1130
eGFR (ml/min/1.73m^2^)	0.97 (0.96–0.98)*	< 0.001	0.99 (0.97–1.00)*	0.032	155/1069
Fasting plasma glucose (mmol/l)	1.24 (1.09–1.41)*	0.001	1.20 (1.07–1.35)*	0.003	144/1088
2 h plasma glucose (mmol/l)	1.16 (1.07–1.26)*	< 0.001	1.12 (1.03–1.22)*	0.006	142/1082
Diabetes at baseline	4.81 (3.08–7.52)	< 0.001	3.47 (2.20–5.45)	< 0.001	161/1130
BMI (kg/m^2^)	1.10 (1.06–1.13)*	< 0.001	1.08 (1.05–1.12)*	< 0.001	160/1127
Systolic blood pressure (mmHg)	1.03 (1.02–1.03)*	< 0.001	1.02 (1.01–1.03)*	< 0.001	159/1120
Diastolic blood pressure (mmHg)	1.03 (1.01–1.04)*	< 0.001	1.03 (1.02–1.05)*	< 0.001	159/1120
Hypertension at baseline	2.75 (1.99–3.81)	< 0.001	2.29 (1.64–3.19)	< 0.001	161/1130
Serum albumin (g/l)	0.96 (0.92–1.00)*	0.029	0.97 (0.93–1.02)*	0.203	143/1029
Plasma total cholesterol (mmol/l)	1.05 (0.89–1.22)*	0.576	0.94 (0.80–1.10)*	0.445	159/1125
Plasma HDL-cholesterol (mmol/l)	0.57 (0.37–0.89)*	0.013	0.58 (0.37–0.93)*	0.023	158/1120
Plasma LDL-cholesterol (mmol/l)	1.00 (0.83–1.20)*	0.996	0.89 (0.74–1.07)*	0.200	147/1097
Plasma triglycerides (mmol/l)	1.27 (1.13–1.42)*	< 0.001	1.27 (1.12–1.44)*	< 0.001	159/1123
Smoking	0.72 (0.52–0.99)	0.042	0.86 (0.60–1.24)	0.862	160/1124

Cox proportional hazards model. Abbreviations: HR, hazard ratio; CI, confidence interval; N/A, results not applicable

eGFR, estimated glomerular filtration rate; BMI, body mass index; HDL, high-density lipoprotein; LDL, low-density lipoprotein

*HR for one-unit increase in continuous variables

Event numbers differ due to missing covariate data

In a multivariable model (Table 3) higher age and higher BMI at baseline, and the presence of baseline diabetes and hypertension remained statistically significant and independently associated with an increased risk of incident CKD. Lower baseline eGFR showed a trend toward association with incident CKD, which was not statistically significant. Several sensitivity analyses were performed (Supplementary files 4–6). Fine-Gray competing risk model was performed with death as a competing risk for incident CKD. The results were broadly consistent with the Cox regression model, but in the competing risk model the association of BMI with incident CKD was not statistically significant. Sex-stratified results of Cox regression models are presented in Supplementary files 7–8.

**Table 3:** Association between risk factors and incident chronic kidney disease in the multivariable adjusted model

Number of events/participants	154/1066	***P*** **-value**
**Factor**	**HR (95% CI)**	
Sex (male)	1.20 (0.87–1.66)	0.262
Age (years)	1.08 (1.05–1.11)*	< 0.001
eGFR (ml/min/1.73m^2^)	0.99 (0.98–1.00)*	0.159
BMI (kg/m^2^)	1.04 (1.00–1.08)*	0.031
Diabetes at baseline	3.17 (1.91–5.28)	< 0.001
Hypertension at baseline	1.90 (1.32–2.70)	< 0.001

Cox proportional hazards model. Abbreviations: HR, hazard ratio; CI, confidence interval; eGFR, estimated

glomerular filtration rate; BMI, body mass index

*HR for one-unit increase in continuous variables

### Prevalence of CKD

Table 4 presents the progression of CKD manifestations across the study phases. The proportion of individuals with CKD increased from 3.1% at baseline to 10.9% at the 10-year follow-up, and to 21.8% at the 22-year follow-up. At baseline and at 10-year follow-up, the prevalence of CKD stage 3 was the highest, while at 22-year follow-up the prevalence of CKD stage 2 had become the most common. The proportion of individuals with proteinuria also increased during the study, from 1.3% at baseline to 3.8% at 10 years and to 13.3% at 22 years. The prevalence of CKD steadily increased with age, from 1.4%, 3.1–5.2%, 6.0–11.3%, 21.0–23.0% and 45.2% in participants aged 40–49 years, 50–59 years, 60–69 years, 70–79 years, and ≥ 80 years, respectively (Supplementary file 9).

**Table 4:** Chronic kidney disease at baseline and follow-ups by different definition methods

	Baseline	10-year follow-up	22-year follow-up
	Number (%)	Number (%)	Number (%)
eGFR < 60 ml/min/1.73m^2^, proteinuria or CKD diagnosis	35 (3.1%)	109 (10.9%)	145 (21.8%)
Missing values	19 (1.7%)	4 (0.4%)	0
Proteinuria corresponding ACR ≥ 3 mg/mmol	11 (1.3%)	27 (3.8%)	86 (13.3%)
Missing values	273 (23.9%)	289 (28.8%)	18 (2.7%)
eGFR decline ≥ 40%	N/A	29 (3.0%)	39 (6.2%)
Missing values	N/A	34 (3.4%)	36 (5.4%)
CKD stages according to eGFR:			
1. ≥ 90 ml/min/1.73m^2^	7 (20.0%)	7 (6.4%)	9 (6.2%)
2. 60–89 ml/min/1.73m^2^	10 (28.6%)	17 (15.6%)	61 (42.1%)
3. 30–59 ml/min/1.73m^2^	17 (48.6%)	79 (72.5%)	59 (40.7%)
4. 15–29 ml/min/1.73m^2^	1 (2.9%)	3 (2.8%)	3 (2.1%)
5. <15 ml/min/1.73m^2^ or on dialysis	0	0	6 (4.1%)
CKD defined only by diagnostic codes or proteinuria	0	3 (2.8%)	7 (4.8%)

Sample sizes vary by laboratory test and follow-up time point due to missing data

Abbreviations: ACR, urine albumin-to-creatinine ratio; eGFR, estimated glomerular filtration rate; CKD, chronic kidney disease; N/A, results not applicable

Since CysC measurements were available only at the 22-year follow-up, the CKD-EPI equation incorporating both SCr and CysC could be applied at a single timepoint only. The total number of participants with CKD was fairly similar across the estimation method used, and no differences were observed in the prevalence of CKD stages 4–5. However, the distribution of CKD stages 1–3 varied across methods (Supplementary file 10).

Awareness of CKD was consistently very low across all study phases. Based on the questionnaires, only 3 (8.6%) of participants with CKD were aware of their condition at baseline, 7 (6.4%) at 10-year follow-up and 6 (4.1%) at the 22-year follow-up.

### Mortality

During the follow-up period, 243 participants (21.2%) died. Men had a 2.79-fold risk of death compared with women (95% CI 2.11–3.68). After adjusting for age and sex, the following baseline variables were statistically significantly associated with increased mortality risk: smoking, higher fasting and 2-hour plasma glucose in OGTT, higher BMI, systolic blood pressure and plasma triglycerides, and the presence of proteinuria, and diabetes and hypertension status (Supplementary file 11).

In an age-adjusted time-dependent Cox proportional hazards model, incident CKD was associated with a 2.31-fold risk of mortality (95% CI 1.54–3.48) (Table 5). The multivariable model included the following baseline covariates that were statistically significant in age- and sex-adjusted analysis: smoking, BMI, and diabetes and hypertension status. The mortality risk associated with incident CKD did not differ between women and men, indicating no statistically significant sex interaction in mortality risk.

Proteinuria, analyzed as a time-dependent variable, was associated with a 2.75-fold risk of death in the age-adjusted analysis (95% CI 1.64–4.60). The mortality risk associated with proteinuria adjusted for age was higher in women than in men (HR 5.51 vs. 1.68), and the difference between sexes remained statistically significant after multivariable adjustment. A ≥ 40% decline in eGFR, also analyzed as a time-dependent variable, was associated with a 2.37-fold increased risk of mortality in the age adjusted analysis (95% CI 1.32–4.26), and the association remained statistically significant in the multivariable model.

**Table 5:** Associations between incident chronic kidney disease, proteinuria, eGFR decline and mortality

Factor	Unadjusted analysis	*P*-value	*P* for interaction (for sex)	Age-adjusted analysis	*P*-value	*P* for for interaction (for sex)	Multivariable^a^ adjustment	*P*-value	*P* for interaction (for sex)
	HR (95% CI)			HR (95% CI)			HR (95% CI)		
Number of events/ participants	237/1130			237/1130			232/1121		
**Incident CKD**	2.91 (1.94–4.37)	< 0.001	0.513	2.31 (1.54–3.48)	< 0.001	0.435	1.61 (1.04–2.49)	0.032	0.332
men	2.59 (1.58–4.26)	< 0.001		2.02 (1.23–3.34)	0.006		1.52 (0.88–2.63)	0.134	
women	3.46 (1.70–7.08)	< 0.001		2.71 (1.32–5.55)	0.007		1.88 (0.89–4.00)	0.099	
**Proteinuria**	3.50 (2.10–5.84)	< 0.001	0.072	2.75 (1.64–4.60)	< 0.001	0.029	1.46 (0.84–2.55)	0.178	0.037
men	2.29 (1.24–4.23)	0.008		1.68 (0.90–3.14)	0.101		1.09 (0.55–2.16)	0.797	
women	6.16 (2.45–15.50)	< 0.001		5.51 (2.16–14.01)	< 0.001		2.77 (1.03–7.44)	0.044	
**eGFR decline 40%**	2.71 (1.51–4.87)	< 0.001	0.495	2.37 (1.32–4.26)	0.004	0.366	2.24 (1.24–4.04)	0.008	0.226
men	3.68 (1.80–7.53)	< 0.001		3.55 (1.73–7.26)	< 0.001		3.57 (1.72–7.44)	< 0.001	
women	2.42 (0.87–6.71)	0.090		2.01 (0.72–5.57)	0.181		1.57 (0.56–4.45)	0.394	

Time-dependent Cox proportional hazards model. Abbreviations: eGFR, estimated glomerular filtration rate; HR, Hazard ratio; CI, Confidence interval;

CKD, chronic kidney disease

^a^Adjustment for baseline age, smoking, body mass index, diabetes, and hypertension

Event numbers differ due to missing covariate data

## Discussion

Our study provides a long-term evaluation of the cumulative incidence of CKD in the general population. To our knowledge, no previous studies have concurrently assessed the development of proteinuria and a ≥ 40% decline in eGFR over an extended follow-up using a similar population-based study design. The high participation rate and the use of data from healthcare registries throughout the study period strengthen the generalizability and comprehensiveness of our findings. Over the 22-year follow-up period, incident CKD was detected in 14.1% of participants, and risk factors for incident CKD observed in this study - such as hypertension, diabetes and obesity - were consistent with previously established predictors. Incident CKD was strongly associated with increased mortality risk. Proteinuria was associated with mortality in the time-dependent-analysis, although the association was not statistically significant in the multivariable model. The magnitude of the association was similar to that observed for a ≥ 40% reduction in eGFR, although these estimates were derived from separate models, and cannot be directly compared. It is possible that the adjustment for potential mediators, such as diabetes and hypertension, may have led to some degree of overadjustment, resulting in an underestimation of the true effect of proteinuria. Therefore, further studies are needed to confirm whether there is a difference between mortality risk associated with proteinuria and a ≥ 40% reduction in eGFR. Although overall mortality was higher in men, the mortality risk associated with incident CKD was not different between sexes.

Our study demonstrates that the incidence and prevalence of CKD stages 1–5 among middle-aged Finns are broadly comparable to estimates reported in other European populations. To date, most epidemiological studies of CKD have focused primarily on stages 3–5, end-stage kidney disease, or kidney replacement therapy. However, more inclusive population-level data are emerging. For example, an Icelandic study reported an overall age-standardized CKD prevalence of 5.12% in men and 6.75% in women, with a mean annual age-standardized incidence of 649 men and 694 women per 100 000 [13]. A Japanese community-based population study with a 10-year follow-up reported a 19.2% prevalence of newly developed CKD [14]. Data from the Framingham Offspring Study estimated the cumulative lifetime risk of CKD from age 50 to 90 years at 41.3% [15]. A Swedish study based on healthcare data found a CKD prevalence of 6.1% [16], and a recent meta-analysis including 119 studies and over 29 million participants worldwide reported a global prevalence of CKD stages 1–5 as 13.0%, which was similar in males and females, and prevalence of stages 3–5 as 6.6%, (7.5% in men and 6.4% in women) [2]. Moreover, a recent German population-based study demonstrated a 3.4% incidence of new-onset decreased eGFR, a 6.9% incidence of new-onset proteinuria and a 1.4% incidence of both findings during a 5-year follow-up period [17].

In our study, several variables were statistically significantly associated with a higher risk of incident CKD, including elevated blood pressure, markers of diabetes and obesity. These findings are consistent with previous literature, as hypertension and diabetes are well-established primary risk factors for CKD, and screening for CKD is routinely recommended for individuals with these conditions [18]. In addition, numerous studies have demonstrated a link between obesity and both the onset and progression of CKD [19]. Smoking has also been identified as an independent risk factor for incident CKD and is associated with an increased mortality among individuals with CKD [20]. Interestingly, in our study, smoking was not associated with incident CKD. One possible explanation for this counterintuitive finding is that individuals with a heavier burden of cardiovascular risk factors – particularly obesity, hypertension, and other cardiovascular risk factors, in combination with smoking – may have experienced premature mortality before CKD could manifest. This interpretation is supported by our observed association between smoking and increased mortality, also among those without diagnosed CKD.

Incident CKD was associated with more than twofold mortality risk. This aligns with numerous previous studies demonstrating that CKD elevates the risk of both cardiovascular and all-cause mortality. Notably, existing evidence suggests that, regardless of CKD stage, individuals with CKD are more than twice as likely to die than progress to ESKD [21]. In our cohort, there was no statistically significant sex difference in mortality associated with incident CKD, although previous studies have demonstrated that among individuals with CKD, men face a greater risk of all-cause mortality than women [22]. In our study, overall mortality was markedly higher in men, who experienced a 2.79-fold risk of death compared with women. In multivariable analyses, proteinuria was associated with higher mortality in women, while mortality associated with a ≥ 40% decline in eGFR was numerically although statistically non-significantly higher in men. The mechanisms underlying sex-related differences in CKD outcomes remain incompletely understood, but for example lower nephron mass in women, the impact of sex hormones, and differences in lifestyle behaviours have been considered as possible mediators [23]. Although our sex-related findings may be partly attributable to chance or residual confounding, they highlight the need for further research into sex-specific determinants of mortality in CKD – particularly regarding the different prognostic impact of proteinuria and eGFR decline between men and women.

Consistent with previous research, awareness of CKD remained low throughout the study period, highlighting the critical need to enhance public and clinical awareness, promote early detection, and ensure timely interventions to mitigate disease progression and associated complications.

### Limitations

Compared with several previous population-based studies, our study included a relatively small number of participants. However, the overall participation rate was high, and the cohort was well balanced in terms of sex distribution. A total of 237 participants were lost to follow-up (i.e., they did not participate in follow-up phases, and laboratory data via registries were not available), but nearly 80% were followed until death or the end of the study. A potential selection bias toward more health-conscious individuals may have affected the results particularly in the follow-up phases. Additionally, healthcare data may overrepresent urine samples collected from individuals with existing diseases or clinical symptoms.

Most proteinuria data were based on a single sample; therefore, we accepted a proteinuria dipstick 1 + result only if it was confirmed with a second sample taken at least 90 days later. While this approach prioritized identifying clinically significant cases, it may have led to the underestimation of milder CKD cases.

Estimation of GFR has many limitations, and can lead to misclassification of CKD, especially if the findings are not confirmed with consecutive measurements. Both SCr and CysC -based equations have several sources of error, which may lead to misinterpretation of kidney function. A decline of ≥ 40% in eGFR is considered a more reliable indicator of meaningful deterioration in renal function than a single cutoff value, because it can be captured even if baseline eGFR is relatively high. However, some individuals with CKD may still have been missed if the eGFR was borderline at baseline, and the subsequent decline was less than 40%. CysC measurements were only available at the 22-year follow-up, but the prevalence estimates were consistent whether the CKD-EPI equation used SCr alone or in combination with CysC.

The exact timing of CKD onset cannot always be determined with complete precision. However, the availability of interim registry-based data substantially reduces the risk of misclassification compared with a study design relying solely on a limited number of discrete observation points.

Despite these limitations, we believe that our study offers a robust and comprehensive estimate of CKD incidence and risk factors in the general population, and that our findings are likely generalizable to other middle-aged or older Western populations.

## Conclusion

Our findings demonstrate that, even in a country with a relatively low incidence of kidney replacement therapy, CKD stages 1–5 are highly prevalent and strongly associated with increased mortality. Importantly, our results highlight the value of urine protein screening not only as a diagnostic tool for CKD, but also as an important prognostic marker for mortality risk. These findings underscore the critical need for early detection and risk-stratification strategies in primary care settings to identify individuals at elevated risk and guide timely interventions.

## Supplementary Information

Below is the link to the electronic supplementary material.

Supplementary Material 1: Supplementary File 1: Questionnaires used in the Savitaipale Study.

Supplementary Material 2: Supplementary File 2: Laboratory methods.

Supplementary Material 3: Supplementary File 3: Comparison of clinical and laboratory variables at baseline by sex; with baseline or incident chronic kidney disease (CKD) and without CKD.

Supplementary Material 4: Supplementary file 4: Association between risk factors and incident chronic kidney disease in the multivariable adjusted model. Cox regression model stratified with baseline diabetes status, and with fasting glucose instead of baseline diabetes status.

Supplementary Material 5: Supplementary file 5: Association between risk factors and incident chronic kidney disease in the multivariable adjusted competing risk model.

Supplementary Material 6: Supplementary file 6: Association between risk factors and incident chronic kidney disease in the multivariable adjusted competing risk model II. Competing risk model stratified with diabetes.

Supplementary Material 7: Supplementary file 7: Association between risk factors and incident chronic kidney disease by sex.

Supplementary Material 8: Supplementary file 8: Association between risk factors and incident chronic kidney disease in the multivariable adjusted mode by sex.

Supplementary Material 9: Supplementary File 9: The prevalence of chronic kidney disease (CKD) by age group and examination years.

Supplementary Material 10: Supplementary File 10: Participants with chronic kidney disease by eGFR category at the 22-year follow-up.

Supplementary Material 11: Supplementary File 11: Association between risk factors and mortality adjusted for age and sex.

## Data Availability

The datasets generated and/or analysed are available from the corresponding author upon reasonable request. Single-level data cannot be shared.

## Abbreviations

ACR: Urine albumin-to-creatinine ratio
BMI: body mass index
CI: Confidence interval
CKD: Chronic kidney disease
CKD-EPI: Chronic Kidney Disease Epidemiology Collaboration
cU-Alb: Albumin from night urine
CysC: Cystatin C
DBP: Diastolic blood pressure
eGFR: estimated glomerular filtration rate
ESKD: End-stage kidney disease
HbA1c: Glycated hemoglobin
HDL: High density lipoprotein
HR: Hazard ratio
ICD-10: The International Classification of Diseases, 10th Revision
IQR: Interquartile range
KDIGO: Kidney Disease: Improving Global Outcomes
LDL: Low density lipoprotein
nU-Alb: Albumin from night urine
OGTT: Oral glucose tolerance test
PER: 24-hour urine protein collection
SBP: Systolic blood pressure
SCr: Serum creatinine
Tg: Triglycerides
