# Supplementary material for: Incidence, risk factors, and mortality impact of chronic kidney disease in middle-aged Finns: a 22-year population-based cohort study
Source: BMC Nephrol. 2026 Apr 14;27:329. doi: 10.1186/s12882-026-04970-6 (PMC13202962; doi:10.1186/s12882-026-04970-6)
Supplement: Supplementary file 1 — Supplementary Material 1: Supplementary File 1: Questionnaires used in the Savitaipale Study. [file 12882_2026_4970_MOESM1_ESM.docx]

**SUPPLEMENTARY FILE 1:**

**QUESTIONNAIRES USED IN THE SAVITAIPALE STUDY:**

**Baseline survey:**

**Questionnaire assessing health status and lifestyle habits (English version below)**

**10-year and 22-year follow-up survey:**

**Questionnaire assessing health status and lifestyle habits (same as in baseline survey)**

**Sosio-economic status**

**Physical activity habits**

**Alcohol consumption**

**Sleep**

**Quality of life: SF-36, 15D**

**Beck´s depression scale**

**VF-14 (Visual Function Index)**

**Men: International Index of Erectile Function (IIEF-5)**

**QUESTIONNAIRE:**

The following questions are divided into two parts: those concerning yourself and those concerning your family. Please answer all questions yourself, according to your own understanding. Answer even if, for example, another family member has already provided the family health information, and it feels unnecessary to repeat the same details. When the same information is obtained from several members of the same family, its reliability increases. In this kind of study such information is highly valuable.

Remember: **This is NOT a test. Your answers will not be judged as right or wrong, or good or bad! It is very important that you answer something for every question, if it is possible.**

**INFORMATION ABOUT YOURSELF:**

**Your current height** (centimeters) _____ (estimate if uncertain)

**Your current weight (**kilograms) ______ (estimate if uncertain)

**INFORMATION ABOUT YOUR BIRTH**:

**Your birth weight** **in grams ______** (Even an approximate estimate is helpful – e.g. “under 2 kilograms” is important information, as it indicates that you were small at the time of your birth)

If you would like to describe your birth weight in words, you may write it here:

**Your birth height in centimeters: _____**  (Even approximate estimate is helpful)

If you would like to describe your birth height in words, you may write it here:

**Were you born full-term** – that is, after pregnancy lasting 9 months?

**□**  Yes

**□**  No, I was born prematurely

How many weeks before due date? _____

**□** After the expected due date

How many weeks after due date? _____

Place of birth

**□** Home

**□** Hospital:

Please specify which hospital:

**□** Other place

If “other place”, please specify:

Are you aware of any complications related to your birth, such as prolonged labor, an emergency

caesarean section, or anything else?

**□** No

**□** Yes

If yes, please specify:

Your mother´s last name at the time of your birth:

Your mother´s maiden name:

**QUESTIONS ABOUT YOUR HEALTH:**

Have you ever had any of the following diseases? If your answer is “YES”, circle the number 1 for

disease name listed below. If your answer is “NO”, circle the number 2.

|  | **YES** | **NO** | **DISEASE** | **AGE AT ONSET** |
| --- | --- | --- | --- | --- |
|  | **1** | **2** | **Heart attack** | **xxxxxxxxxxxxxxx** |
|  |  |  | If yes, at what age first time |  |
|  | **1** | **2** | **Chest pain relieved by nitroglycerin** | **xxxxxxxxxxxxxxx** |
|  |  |  | If yes, at what age first time |  |
|  | **1** | **2** | **Stroke** | **xxxxxxxxxxxxxxx** |
|  |  |  | If yes, at what age first time |  |
|  | **1** | **2** | **Intermittent claudication** | **xxxxxxxxxxxxxxx** |
|  |  |  | If yes, at what age first time |  |
|  | **1** | **2** | **Protein in urine** | **xxxxxxxxxxxxxxx** |
|  |  |  | If yes, at what age first time |  |
|  | **1** | **2** | **Eye disease*** | **xxxxxxxxxxxxxxx** |
|  |  |  | If yes, at what age first time |  |
|  | **1** | **2** | **Kidney disease**** | **xxxxxxxxxxxxxxx** |
|  |  |  | If yes, at what age first time |  |
|  | **1** | **2** | **Gallstones** | **xxxxxxxxxxxxxxx** |
|  |  |  | If yes, at what age first time |  |
|  | **1** | **2** | **Gout** | **xxxxxxxxxxxxxxx** |
|  |  |  | If yes, at what age first time |  |
|  | **1** | **2** | **Rheumatoid arthritis** | **xxxxxxxxxxxxxxx** |
|  |  |  | If yes, at what age first time |  |
|  | **1** | **2** | **Thyroid disease** | **xxxxxxxxxxxxxxx** |
|  |  |  | If yes, at what age first time |  |
|  | **1** | **2** | **Pancreatitis** | **xxxxxxxxxxxxxxx** |
|  |  |  | If yes, at what age first time |  |
|  | **1** | **2** | **Hereditary disease** | **xxxxxxxxxxxxxxx** |
|  |  |  | If yes, specify: |  |

***Eye disease: If yes, what kind? Check the options that apply. Check the box .**

**□** Cataract

Surgically treated? □ Yes □ No

**□** Retinal disease

Have you received laser treatment? □ Yes □ No

If yes, your age at the time of laser treatment _____ (years)

□ Other eye disease

Which?

** **Kidney disease: If yes, what kind?** **If you have had multiple conditions, please check all that**

**apply. Check the box.**

**□** Bladder infection

**□** Kidney infection with fever and possible hospitalization (pyelonephritis)

**□** Other kidney disease

Which?

**SURGICAL OPERATIONS:**

Have you undergone any of the following surgeries? If your answer is “YES”, circle the number 1 for any

surgical operation listed below. If your answer is “NO”, circle the number 2.

|  | **YES** | **NO** | **TYPE OF SURGERY** | **AGE AT SURGERY** |
| --- | --- | --- | --- | --- |
|  | **1** | **2** | **Heart bypass surgery** | **xxxxxxxxxxxxxxx** |
|  |  |  | If yes, at what age first time |  |
|  | **1** | **2** | **Other vascular surgery#** | **xxxxxxxxxxxxxxx** |
|  | **1** | **2** | **Gallbladder surgery** | **xxxxxxxxxxxxxxx** |
|  |  |  | If yes, at what age first time |  |
|  | **1** | **2** | **Pancreatic surgery** | **xxxxxxxxxxxxxxx** |
|  |  |  | If yes, at what age first time |  |
|  | **1** | **2** | **Kidney surgery** | **xxxxxxxxxxxxxxx** |
|  |  |  | If yes, at what age first time |  |

**# Other vascular surgery:** If yes, what kind? If you have had multiple operations, please check

all that apply. Check the box.

**□** Varicose vein surgery (lower limbs)

If yes, your age at the time of surgery (years):

**□** Lower limb arterial reconstruction surgery

If yes, your age at the time of surgery (years):

**□** Cerebral vascular surgery

If yes, your age at the time of surgery (years):

**HIGH BLOOD PRESSURE:**

Have you ever had your blood pressure measured? Check the box.

**□** Yes

**□** No **🡪** Proceed to the question **“**BLOOD SUGAR**”**

When was your blood pressure last measured? Check the box.

**□** Less than a week ago

**□** Less than a month ago

**□** Within the last 3 months

**□** Within the last 6 months

**□** Within the last year

**□** Within the last 5 years

**□** More than 5 years ago

Have you ever been told that your blood pressure is elevated or too high? Check the box.

**□** No

**□** Yes 🡪

At what age were you first informed that you had high blood pressure? _____ years

Do you remember what your blood pressure was at that time

The upper (systolic) value _____ mmHg

The lower (diastolic) value ______ mmHg

What is the highest blood pressure that has ever been measured for you?

The upper (systolic) value _____ mmHg

The lower (diastolic) value ______mmHg

When was it measured? (year) 19__

**BLOOD SUGAR:**

Have you ever had your blood sugar measured? Check the box.

**□** Yes

**□** No 🡪 Proceed to the question “CHOLESTEROL”

When was your blood sugar last measured?

**□** Less than a week ago

**□** Less than a month ago

**□** Within the last 3 months

**□** Within the last 6 months

**□** Within the last year

**□** Within the last 5 years

**□** More than 5 years ago

The reason for the measurement was

**□** It was a part of a routine health check-up

**□** Family history of diabetes

**□** My blood sugar levels had been found to be abnormal previously

**□** It was tested in connection with another illness

Have you ever been told that your blood sugar is elevated or too high?

**□** No

**□** Yes 🡪

At what age were you first informed that you had high blood sugar? _____ years

Do you remember what your blood sugar was at that time: _______ mmol/l

**CHOLESTEROL:**

Have you ever had your cholesterol measured? Check the box.

**□** Yes

**□** No **🡪** Proceed to the question **“**OVERWEIGHT**”**

When was your cholesterol last measured?

**□** Less than a week ago

**□** Less than a month ago

**□** Within the last 3 months

**□** Within the last 6 months

**□** Within the last year

**□** Within the last 5 years

**□** More than 5 years ago

The reason for the measurement was

**□** It was part of a routine health check-up

**□** Family history of heart disease

**□** My cholesterol levels had been found to be elevated previously

**□** It was tested in connection with another illness

Which illness? _____________

Have you ever been told that your cholesterol is elevated or too high?

**□** No

**□** Yes 🡪

At what age were you first informed that you had elevated cholesterol? _____ years

Do you remember what your cholesterol was at that time: _______ mmol/l

**OVERWEIGHT:**

Have you ever been advised by a doctor to lose weight? Check the box.

**□** No

**□** Yes 🡪

How old were you at that time? ____ years

Approximate weight at that time: _____ kg

What explanation did you receive for why losing weight was important?

**□** My blood pressure was too high, and losing weight would help with it

**□** My blood sugar was too high, and losing weight would help with it

**□** My cholesterol was too high, and losing weight would help with it

**□** Suspected coronary artery disease and weight loss recommended as part of treatment

**□** Other reason, please specify:

Number of times you have attempted weight loss:

______ times (enter 0 if never)

**SMOKING:**

Do you smoke?

**□** I have never smoked 🡪 Proceed to the question “OCCUPATION”

**□** I used to smoke but have quit

I quit smoking **____** years ago

**□** I currently smoke: Check the box.

**□** Less than 5 cigarettes / day

**□** 5-10 cigarettes / day

**□** 10-20 cigarettes / day

**□** More than 20 cigarettes / day

**OCCUPATION:**

Are you currently employed?

**□** Yes

Please write your occupation here

**□** No 🡪

The reason for not being employed:

**□** I am on disability pension due to illness

**□** I am retired due to age

**□** I am unemployed

**MEDICATION:**

Do you have medication for high blood pressure?

**□** No

**□** Yes 🡪

Write here the name of the medication and your daily dosage:

Do you have medication for elevated blood sugar?

**□** No

**□** Yes 🡪

Write here the name of the medication and your daily dosage:

To be completed by the nurse:

□ Dietary treatment

□ Dietary treatment + oral medication

□ Dietary treatment + oral medication + insulin

□ Dietary treatment + insulin

□ No treatment

Do you have medication for heart disease or chest pain?

**□** No

**□** Yes 🡪

Write here the name of the medication and your daily dosage:

Do you have medication for asthma?

**□** No

**□** Yes 🡪

Write here the name of the medication and your daily dosage:

Do you have any other medication?

**□** No

**□** Yes 🡪

Write here the name of the medication and your daily dosage:

Question for women:

Do you use estrogen therapy for birth control or menopause?

**□** No

**□** Yes 🡪

Write here the name of the medication and your daily dosage:
