# Supplementary material for: Incidence, risk factors, and mortality impact of chronic kidney disease in middle-aged Finns: a 22-year population-based cohort study
Source: BMC Nephrol. 2026 Apr 14;27:329. doi: 10.1186/s12882-026-04970-6 (PMC13202962; doi:10.1186/s12882-026-04970-6)
Supplement: Supplementary file 2 — Supplementary Material 2: Supplementary File 2: Laboratory methods. [file 12882_2026_4970_MOESM2_ESM.docx]

SUPPLEMENTARY FILE 2:

**Laboratory methods:**

Serum creatinine, serum albumin and serum cystatin C were analysed from frozen samples (baseline samples stored in -22°C, follow-ups in -80°C).

All the laboratory measurements were made in Vita Laboratoriot (www.vita.fi)

**Creatinine:**

CREP 2 (Creatinine Plus ver.2, Roche diagnostics)

- Enzymatic method based on the conversion of creatinine with the aid of creatininase, creatinase and sarcosine oxidaxe to glycine, formaldehyde and hydrogen peroxide.

**Cystatin C:**

CYSC2 (Tina-quant Cystatin C Gen.2, Roche diagnostics)

- Particle enhanced immunoturbidimetric assay

**Albumin:**

ALB2 (Albumin Gen.2, Roche diagnostics)

- Colorimetric assay
